# Supplementary material for: Multiple host targets of Pseudomonas effector protein HopM1 form a protein complex regulating apoplastic immunity and water homeostasis
Source: bioRxiv. 2023 Aug 1:2023.07.31.551310. Preprint. [Version 1] doi: 10.1101/2023.07.31.551310 (PMC10418078; doi:10.1101/2023.07.31.551310)
Supplement: 1 [file NIHPP2023.07.31.551310V1-supplement-1.pdf]

bioRxiv preprint doi: https://doi.org/10.1101/2023.07.31.551310; this version posted August 1, 2023. The copyright holder for this preprint (which was not certified by peer review) is the author/funder, who has granted bioRxiv a license to display the preprint in perpetuity. It is made available under aCC-BY 4.0 International license.

Supporting Information

Multiple host targets of *Pseudomonas* effector protein HopM1 form a protein complex regulating apoplastic immunity and water homeostasis

Kinya Nomura[1,2], Lori Alice Imboden[3], Hirokazu Tanaka[4] and Sheng Yang He[1,2*]

List of Content:

Supporting information Table (Table S1)
Supporting information Figures (Figs. S1 to S5)

Table S1. Arabidopsis proteins pulled down with GFP *in vivo*.

Figure S1. HopM1 weakly interacts with MIN13 (an ARF-GEF) in Y2H assay.

HopM1-N and MIN13 were expressed from pGILDA (BD fusion) and pB42AD (AD fusion), respectively. Yeast cultures were spotted and grown on minimal medium containing galactose and X-gal. A blue color indicates interaction, whereas a white color indicates no interaction. AD-MIN7 was used as a positive control.

Figure S2. Gene families of the components of the MIN7 complex.

(A) HLB1 does not share significant sequence similarity to any protein of known function. BlastP search against the nr database was performed using HLB1 as a query. The names and BlastP scores of the top ten protein sequences are displayed.

(B) A phylogenetic tree of ARF-GEF proteins constructed using CLUSTAL 2.1 Multiple Sequence Alignments.

(C) A phylogenetic tree of 14-3-3 proteins constructed using CLUSTAL 2.1 Multiple Sequence Alignments.

(D) A phylogenetic tree of ARF GTPase proteins constructed using CLUSTAL 2.1 Multiple Sequence Alignments.

Figure S3. Co-IP analysis of MIN7 and MIN10 proteins.

35S::MIN10-HA/Col-0 and Col-0 plants were treated with 1µM flg22 for 6 h. Total Arabidopsis extracts (Input) and immunoprecipitates (HA-agarose IP) were separated on an SDS-PAGE gel and subjected to immunoblot analysis. MIN7 and MIN10-HA were detected by MIN7- and HA-specific antibodies, respectively.

Figure S4. Disease symptoms on *min7*, *hlb1*, *min7/hlb1* or Col-0 plants.

bioRxiv preprint doi: https://doi.org/10.1101/2023.07.31.551310; this version posted August 1, 2023. The copyright holder for this preprint (which was not certified by peer review) is the author/funder, who has granted bioRxiv a license to display the preprint in perpetuity. It is made available under aCC-BY 4.0 International license.

Plants were inoculated by dipping with $1x10^8$ cfu/ml bacteria and immediately covered with a clear plastic dome to maintain high humidity. Disease symptoms (chlorosis and necrosis) in Col-0 and mutant plants were recorded at day 4.

Figure 5. Bacterial multiplication in Arabidopsis mutant plants affected in the components of the MIN7 complex.

(A) to (C) Arabidopsis plants were dip-inoculated with the *Pst* DC3000, ΔEM mutant, and the *hrcC* mutant at1×10^8 cfu/ml and immediately covered with a clear plastic dome to maintain high humidity. Bacterial populations (mean ± SEM; n=4 leaf samples) in leaves were determined at day 4 post dip inoculation.

A

Description	Max Score	Total Score	Query Coverage	E-Value	Max Ident	Accession
tetratricopeptide repeat domain-containing protein (HLB1)	1156	1156	100%	0.0	100%	NP_199010.1
mitochondrial import receptor subunit TOM20-3	35.8	67.0	27%	0.035	28%	NP_189344.1
putative UDP-N-acetylglucosamine--peptide N-acetylglucosaminyltransferase SPINDLY	37.0	102	34%	0.041	27%	NP_187761.1
mitochondrial import receptor subunit TOM20-1	35.4	35.4	24%	0.055	24%	NP_189343.1
putative UDP-N-acetylglucosamine--peptide N-acetylglucosaminyltransferase SEC	36.2	36.2	28%	0.068	27%	NP_187074.1
mitochondrial import receptor subunit TOM20-2	33.9	33.9	12%	0.17	30%	NP_174059.2
basic helix-loop-helix domain-containing protein	33.5	33.5	13%	0.24	34%	NP_193829.2
Lysine-specific histone demethylase 1-1	33.9	33.9	9%	0.31	39%	NP_176471.1
Calcium-binding tetratricopeptide repeat-containing protein	33.5	33.5	19%	0.44	24%	NP_180804.1
calmodulin-domain protein kinase cdpk isoform 2	32.7	32.7	14%	0.64	40%	NP_187677.1

B

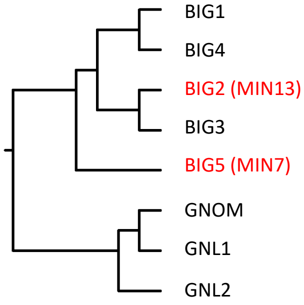

C

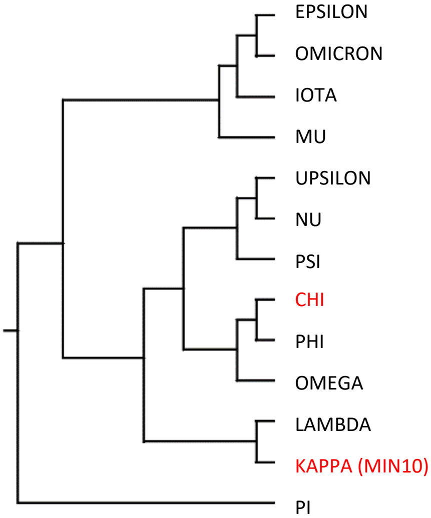

D

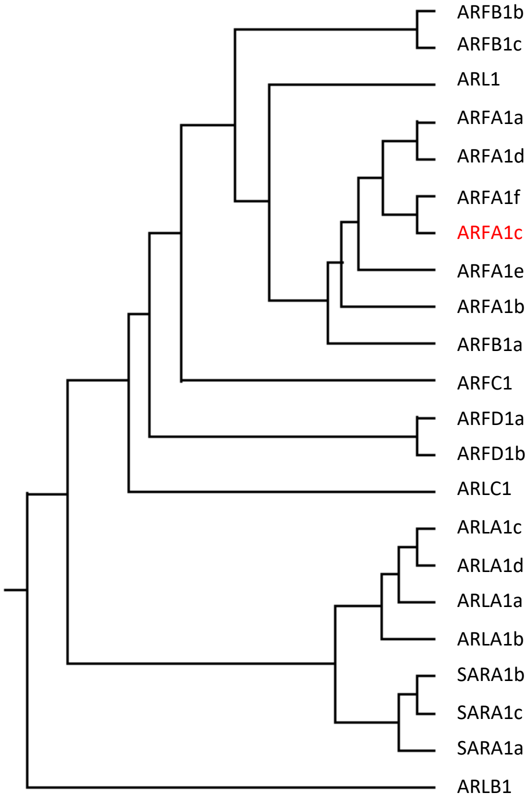

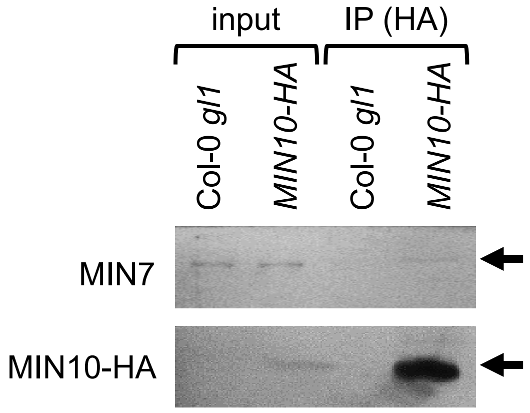
input
IP (HA)
Col-0 gl1
MIN10-HA
Col-0 gl1
MIN10-HA
MIN7
MIN10-HA

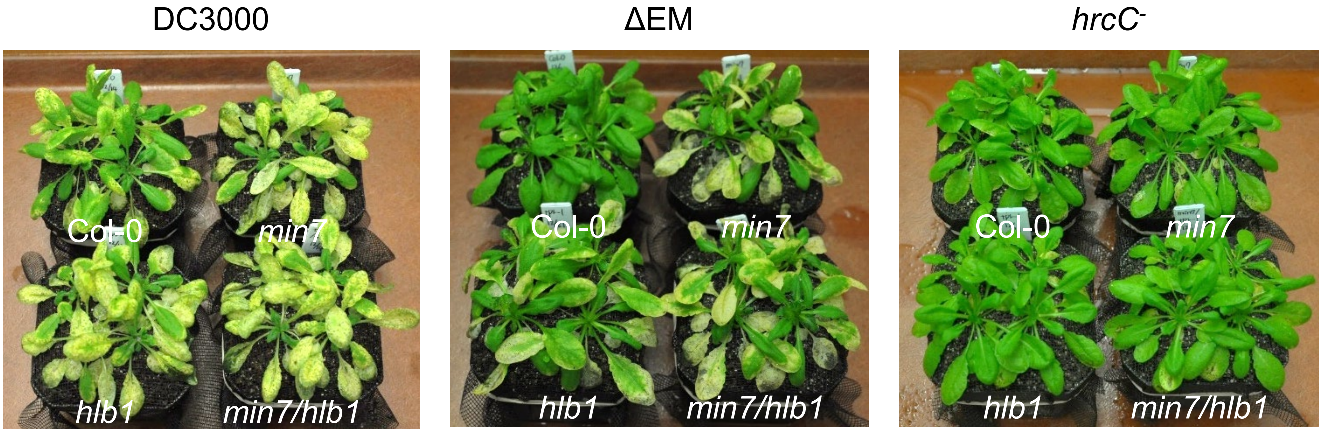
DC3000
ΔEM
hrcC-
Col-0
min7
hlb1
min7/hlb1
Col-0
min7
hlb1
min7/hlb1
Col-0
min7
hlb1
min7/hlb1

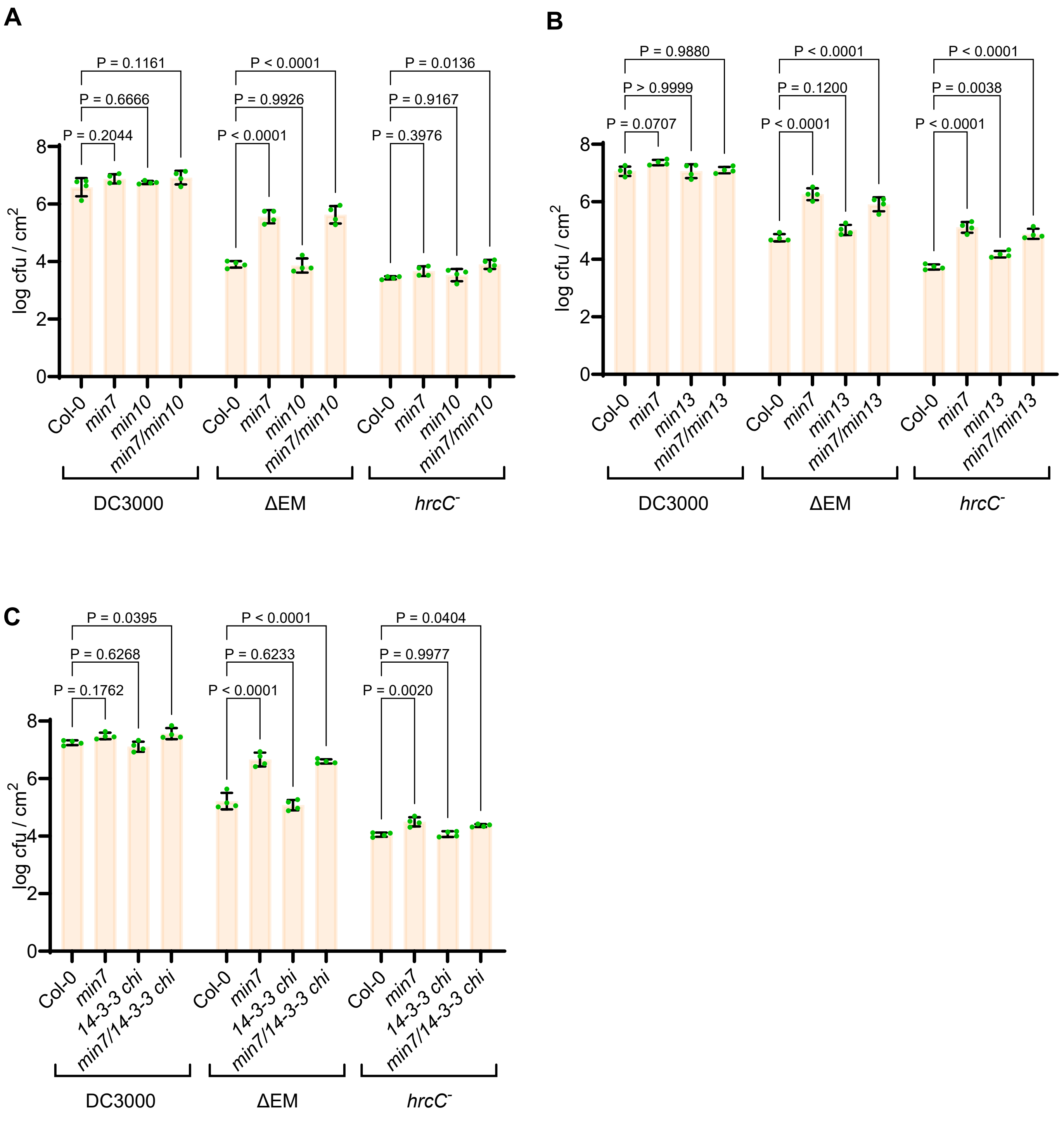
A
P = 0.1161
P = 0.6666
P = 0.2044
P < 0.0001
P = 0.9926
P < 0.0001
P = 0.0136
P = 0.9167
P = 0.3976
log cfu / cm2
8
6
4
2
0
Col-0
min7
min10
min7/min10
DC3000
ΔEM
hrcC-
B
P = 0.9880
P > 0.9999
P = 0.0707
P < 0.0001
P = 0.1200
P < 0.0001
P < 0.0001
P = 0.0038
P < 0.0001
log cfu / cm2
Col-0
min7
min13
min7/min13
DC3000
ΔEM
hrcC-
C
P = 0.0395
P = 0.6268
P = 0.1762
P < 0.0001
P = 0.6233
P < 0.0001
P = 0.0404
P = 0.9977
P = 0.0020
log cfu / cm2
Col-0
min7
14-3-3 chi
min7/14-3-3 chi
DC3000
ΔEM
hrcC-
